# Epigenetic Regulation of Divergent Co-Expression Between Whole-Genome Duplication and Transposed Duplication Genes and Its Impact on Catechin Accumulation in *Camellia sinensis*

**DOI:** 10.3390/genes17080898

**Published:** 2026-07-30

**Authors:** Shuaibin Lian, Huajin Feng, Haojie Hou, Liang Zhang, Youchao Tu, Ke Gong, Wei Zhang

**Affiliations:** 1 College of Physics and Electronic Engineering, Xinyang Normal University, Xinyang 464000, China; 18258101472@163.com (H.F.); haojie12210202@163.com (H.H.); 19836313652@163.com (L.Z.); tyc3216@163.com (Y.T.); gongkexynu@163.com (K.G.); 2 College of Life Sciences, Xinyang Normal University, Xinyang 464000, China

**Keywords:** whole-genome duplication, transposed duplication, epigenetics, chromatin accessibility, DNA methylation, transcription factors, catechins

## Abstract

Background/Objectives: Whole-genome duplication (WGD) and transposed duplication (TRD) are two principal evolutionary drivers of plant genome expansion, yet the molecular mechanisms underlying their divergent co-expression patterns remain poorly characterized. Methods: In this study, based on the reference genome of tea plant (*Camellia sinensis* ‘Yunkang 10’, YK10), we integrated publicly available transcriptomic, ATAC-seq, H3K27ac ChIP-seq, whole-genome bisulfite sequencing (WGBS), and SNP data from eight tissues and performed a multi-layered analysis of co-expression divergence across 4071 WGD and 10,174 TRD gene pairs. Results: WGD gene pairs exhibited significantly higher co-expression rates (44.3%) than TRD pairs (33.0%), with gene length and sequence similarity jointly promoting co-expression. Chromatin accessibility and H3K27ac modification were each positively correlated with expression and co-expression; however, under equivalent chromatin accessibility conditions, TRD gene expression remained systematically attenuated, and this reduced expression state was significantly associated with elevated levels of CG, CHG, and CHH methylation, suggesting that these epigenetic marks may collectively participate in the transcriptional repression of TRD genes. Promoter-proximal SNPs exerted disproportionately deleterious effects on TRD co-expression, demonstrating that the combined effects of genetic variation and epigenetic modifications are associated with enhanced transcriptional divergence. Weighted gene co-expression network analysis (WGCNA) revealed that WGD modules showed significant associations with EC, GC, and EGC accumulation, whereas WRKY and bHLH transcription factors in the TRD MEblue module exhibited strong associations with EGCG and ECG. Conclusions: This study systematically characterizes multi-omics association patterns related to duplicate gene co-expression divergence, providing insights into potential hierarchical regulation. It offers mechanistic clues for catechin metabolic regulation and provides candidate targets for metabolite-directed breeding.

## 1. Introduction

Gene duplication is one of the most important mechanisms driving plant genome expansion and the evolution of novel biological functions [1,2]. Whole-genome duplication (WGD) simultaneously produces copies of all genes through genome-wide polyploidization, and approximately 75% of angiosperm species have ancestral WGD events in their evolutionary history [3,4]. Transposed duplication (TRD), by contrast, relocates parental genes to new genomic positions via RNA-mediated retroposition or DNA-mediated transposition, generating gene pairs consisting of ancestral loci and new insertion sites [5,6]. These two duplication modes differ fundamentally in their genomic positional effects: WGD-retained copies reside within the same chromatin topological environment as their parental genes and are subject to strong dosage balance constraints [7], whereas TRD copies are “transplanted” to entirely new genomic locations, disengaging from the ancestral cis-regulatory landscape and acquiring the structural basis for expression divergence. Comparative studies in Arabidopsis thaliana and Oryza sativa have demonstrated that WGD genes generally exhibit higher conservation in expression levels and patterns than TRD genes [8,9]; however, the molecular mechanisms driving the divergent co-expression fates of these two duplicate types—specifically how chromatin accessibility, DNA methylation, and genetic variation act cooperatively—have not been systematically elucidated in polyploid crop genomes.

Tea plant (*C. sinensis*) leaves are the raw material for the most widely consumed non-alcoholic beverage worldwide [10]. The catechin compounds enriched in tea leaves (comprising 18–36% of dry weight) not only impart the distinctive flavor of tea but also confer significant health-promoting effects, including antioxidant activity [11,12]. Multiple high-quality chromosome-level genome assemblies of tea plant cultivars have been released in recent years, including *C. sinensis* var. *assamica* (CSA, YK10), *C. sinensis* var. *sinensis* (CSS, Shuchazao, Biyun, Longjing 43, Tieguanyin, Huangdan), and ancient tea trees (DASZ). Genomic analyses have indicated that tea plant underwent a WGD event approximately 30–40 million years ago [13,14], and the genome harbors abundant TRD genes [15]. The co-occurrence of ancient WGD and active TRD events in tea plant, combined with deeply characterized catechin metabolic pathways, quantifiable phenotypes, and publicly available multi-tissue transcriptomic, epigenomic, and three-dimensional genomic datasets [16], makes tea plant an ideal system for systematically dissecting the differential regulatory mechanisms of these two duplicate gene types.

Chromatin accessibility profiled by ATAC-seq marks open regulatory regions accessible to transcription factors across the genome [17]; H3K27ac acetylation modification serves as a molecular signature of active enhancers and promoters [18]; and DNA methylation in CG, CHG, and CHH sequence contexts plays critical roles in transposable element silencing and gene expression repression in plants [19]. Whether these regulatory layers act in a differential manner on WGD versus TRD duplicate genes constitutes a key scientific question for understanding the divergence of duplicate gene transcriptional fates—a question that has yet to receive a systematic answer in the literature.

Using the YK10 tea plant genome as a research framework, the present study aims to address the following core scientific questions: (1) What are the differences in genome-wide co-expression rates between WGD and TRD gene pairs in *C. sinensis*? (2) What structural, epigenetic, and genetic factors drive the divergent co-expression patterns between WGD and TRD gene pairs—specifically, how do gene length, sequence similarity, chromatin accessibility, DNA methylation, and promoter-region SNPs act individually and cooperatively to determine co-expression fates? (3) Through what differential transcription factor regulatory networks do the two duplicate gene types cooperatively drive diversified catechin accumulation? The results of this study will provide new mechanistic insights into the evolutionary dynamics of duplicate genes in plants and offer candidate molecular targets for tea plant catechin quality improvement.

## 2. Materials and Methods

### 2.1. Identification of WGD and TRD Gene Pairs

**Figure 1:**
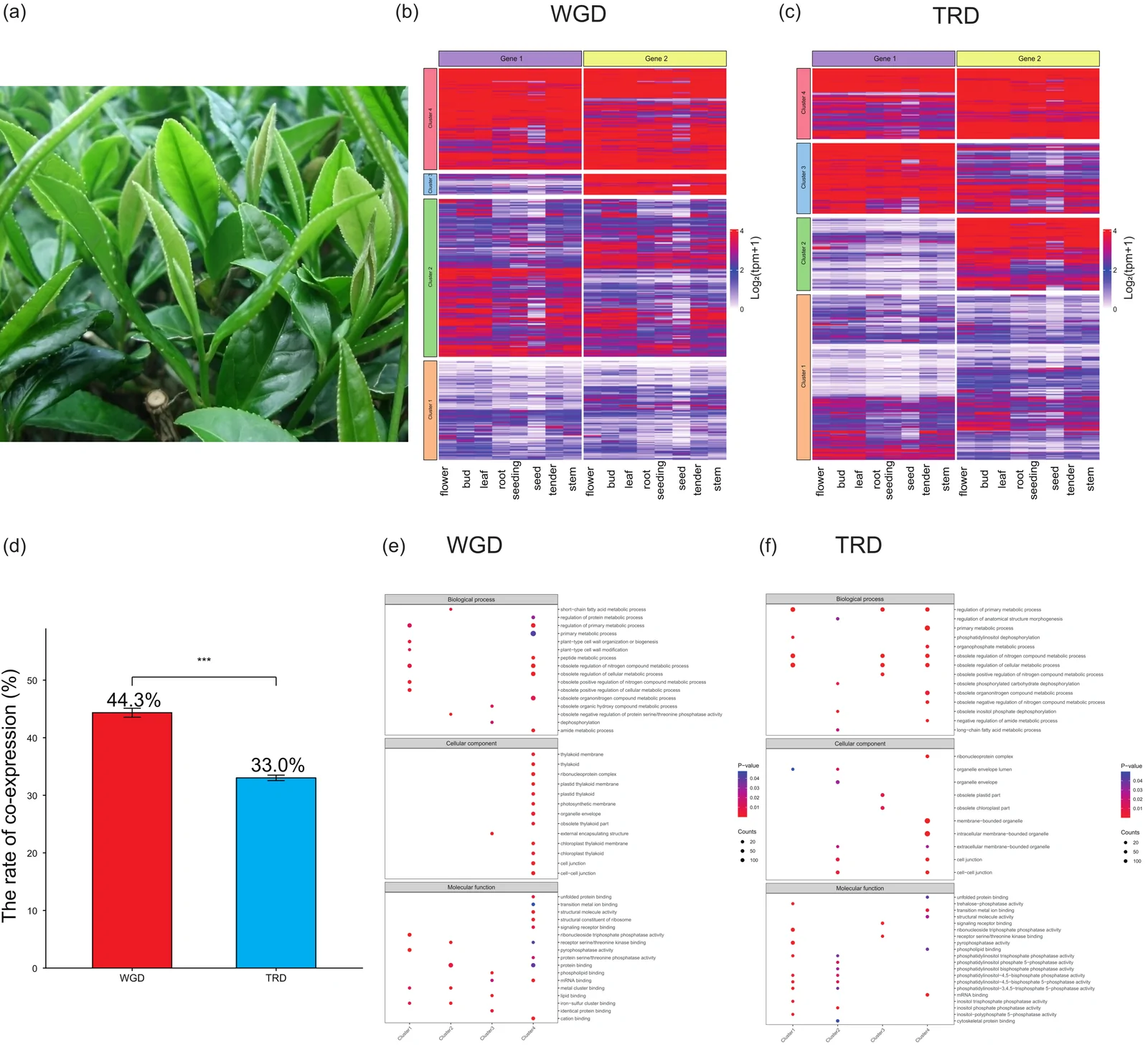
Divergent co-expression patterns of WGD and TRD duplicate gene pairs in *C. sinensis*. (**a**) Photograph of the YK10 tea plant cultivar (http://tpia.teaplants.cn (accessed on 24 July 2026)). (**b**,**c**) Heatmaps of differential expression patterns of WGD and TRD gene pairs across eight tissues, respectively. (**d**) Genome-wide co-expression rate comparison between WGD and TRD gene pairs. (**e**,**f**) GO enrichment analysis of WGD and TRD gene clusters, respectively. Error bars, bootstrapped standard errors. Significance values were determined by the Mann–Whitney U test (*** *p* < 0.001).

The reference genome of tea cultivar YK10 (*Camellia sinensis* var. *assamica*) was used in this study (Figure 1a). YK10 is a widely cultivated large-leaf variety in southwestern China with substantial agronomic value, ensuring the relevance of our findings to major tea germplasm. As the first high-quality reference genome for *C. sinensis*, YK10 has become a benchmark in tea genomics and its latest chromosome-level assembly enabled accurate gene annotation and multi-omics integration here. Duplicate gene pairs were identified following Qiao et al. [20], with Amborella trichopoda as outgroup to distinguish WGD and TRD events (Tables S1 and S2).

### 2.2. Quality Control and Processing of Multi-Omics Data

The multi-omics datasets used in this study were derived from distinct tea cultivars. RNA-seq and SNP data were generated from the ‘Yunkang 10’ (YK10) cultivar, whereas ATAC-seq, H3K27ac ChIP-seq, and WGBS data were obtained from the ‘Tieguanyin’ cultivar. Detailed information on cultivar origin, tissue type, developmental stage, and original publication for each dataset is provided in Table S3. Given that the multi-omics data originated from different cultivars, the integrative analyses presented here should be interpreted as gene/locus-level association integration, rather than sample-matched multi-omics profiling. The SNP data were retrieved from the TeaGVD population database and represent population-level polymorphic sites mapped to the YK10 reference genome, rather than variants called from the same biological samples used for RNA-seq analysis in this study. The processing procedures for each dataset are described in detail below. All datasets used in this study were obtained from public databases (see Data Availability Statement) and evaluated for quality and reliability using multiple approaches. First, RNA-seq data quality was assessed using FastQC, followed by filtering and trimming with fastp using default parameters [21]. Clean reads were aligned to the YK10 reference genome using HISAT2 [22], and gene-level quantification was performed using featureCounts [23]. Gene expression levels were normalized using the TPM method. The processed RNA-seq data exhibited high alignment rates and uniform coverage, indicating accurate transcript quantification. Second, ATAC-seq and H3K27ac ChIP-seq datasets were quality-checked using FastQC and fastp [21]. Clean reads were aligned to the YK10 genome using Bowtie2 [24], and low-quality alignments (Q < 10) were removed using SAMtools [25]. Duplicate reads were filtered with Picard, and peak calling was performed using MACS2 [26]. Peak reproducibility across biological replicates was evaluated with DeepTools, and peaks were successfully annotated to duplicate genes using BEDTools [27], confirming the reliability of chromatin accessibility and histone modification datasets. Third, WGBS raw data were filtered with fastp, reads were aligned to the YK10 genome using the BatMeth2 pipeline [28], and 5-methylcytosine (5mC) sites were identified with methylation levels calculated. Bigwig files were generated using batmeth2_to_bigwig.py, and methylation levels upstream and downstream of peaks were computed with methyGff. Finally, promoter regions (2 kb upstream of the transcription start site) were submitted to the PlantCARE database for cis-regulatory element identification, and transcription factors were predicted using PlantTFDB v5.0 (Table S4) [29,30]. GO annotation was obtained using the eggNOG-mapper database [31].

### 2.3. Randomization Test

A simulation study was performed using the R programming language based on the complete YK10 genome. In each iteration, two genes were randomly selected from the gene category and their Pearson correlation coefficient was calculated from TPM values. The number of gene pairs sampled in each simulation corresponded to the number of duplicate gene pairs within each length category. The proportion of co-expressed gene pairs (gene pairs with Pearson *r* > 0.5 were defined as co-expressed, and the remainder were classified as non-co-expressed) was recorded per run. This process was repeated 100,000 times, and the frequency distribution of co-expression proportions across all iterations was subsequently analyzed.

### 2.4. Weighted Gene Co-Expression Network Construction

Weighted gene co-expression networks were constructed using WGCNA (v4.3.2). To avoid redundancy from genes present in multiple duplicate pairs, a non-overlapping gene set of 7313 WGD and 14,285 TRD genes was retained, together with six catechin traits [14,32]. Network construction used expression data from 78 samples across eight tissues, with tissue-level means as input. Signed networks were built with Pearson correlations; soft-thresholding power = 14 was selected (scale-free R^2^ > 0.9). Minimum module size = 30, merging threshold = 0.25. Modules were visualized with Cytoscape (v3.9.0). As tissue means (not biological replicates) were used, this analysis is exploratory and aims to identify cross-tissue conserved modules. For transcription factor screening, genes in each module were ranked by KME, and the top 50 were selected as core genes. TF family members were annotated via PlantTFDB, without statistical enrichment testing.

### 2.5. Statistical Analysis

Co-expression levels between gene pairs were evaluated using Pearson correlation coefficients (*r*), calculated from log_2_-transformed TPM expression values averaged across 78 samples spanning eight tissue types. To minimize the influence of low-expression genes on correlation estimation, only genes with TPM ≥ 1 in at least one tissue were retained prior to analysis. In gene co-expression studies, *r* > 0.5 is generally considered indicative of a biologically meaningful strong correlation, corresponding to a moderate or greater effect size (R^2^ > 0.25). Accordingly, gene pairs with *r* > 0.5 were defined as co-expressed, whereas those with *r* ≤ 0.5 were classified as non-co-expressed. All statistical analyses were performed using R software (v4.3.2). Differences between two independent groups were assessed using the Mann–Whitney U test (wilcox.test function), with the significance threshold set at *p* < 0.05. The Mann–Whitney U test is a non-parametric method for comparing medians between two independent groups; it does not assume normality and is therefore well suited for skewed or non-normally distributed datasets. To provide robust inference for error bars in bar plots, we employed a bootstrap resampling approach. Specifically, for each group of binary observations, we performed 1000 bootstrap iterations with replacement, each generating a bootstrap sample of the same size as the original dataset. The proportion of positive cases was calculated for each resampled dataset, and the standard deviation of these 1000 proportions was used as the standard error for constructing error bars in the figures.

## 3. Results

### 3.1. WGD and TRD Genes Display Markedly Different Co-Expression Patterns

To systematically characterize the expression patterns and functional divergence of WGD and TRD gene pairs during tea plant development, we performed expression clustering analyses for both gene types based on transcriptomic data from eight tissues. Both gene types were partitioned into four clusters with distinct expression profiles (Figure 1b,c), indicating significant tissue-specific expression divergence in both WGD and TRD genes. A substantial proportion of gene pairs within the same cluster displayed coordinated high expression, suggesting shared transcriptional regulatory mechanisms. At the overall co-expression level, WGD gene pairs (44.3%) showed significantly higher co-expression rates than TRD gene pairs (33.0%; Mann–Whitney U test, *p* < 0.001; Figure 1d), indicating stronger expression coordination between WGD duplicate copies. Across individual clusters, the co-expression rates of WGD gene pairs were consistently higher than those of TRD gene pairs in Clusters 1, 2, and 4 (46.94% vs. 34.76%, 45.45% vs. 25.31%, and 43.84% vs. 34.12%, respectively); however, in Cluster 3, TRD gene pairs exhibited a higher co-expression rate (35.82%) than WGD gene pairs (28.76%). The proportion of WGD gene pairs exhibiting expression divergence (fold change ≥ 2) across tissues (42.94–56.77%; mean 51.3%) was also lower than that of TRD gene pairs (58.50–65.74%; mean 65.5%; Figure S1a,b), further supporting the stronger expression divergence tendency in TRD duplicate copies.

GO enrichment analysis revealed systematic differences in functional specialization between the two duplicate gene types (Figure 1e,f). WGD duplicate genes were primarily enriched in photosynthetic systems and core metabolism: Cluster 1 was significantly enriched in thylakoid membrane, chloroplast thylakoid, and other photosynthetic components, and at the molecular function level in transition metal ion binding, iron-sulfur cluster binding, and ribosomal structural constituents, suggesting central roles in light energy capture, electron transfer, and protein synthesis; Clusters 2 and 3 were co-enriched in primary metabolic processes, protein metabolic regulation, and organic nitrogen compound metabolism, with protein binding and signal receptor binding as primary molecular functions; Cluster 4 was specifically enriched in dephosphorylation, plant-type cell wall modification, and pyrophosphatase activity, indicating key roles in phosphorylation signaling and cell wall remodeling. In contrast, TRD duplicate genes exhibited more pronounced functional specialization while retaining partial core metabolic functions: Cluster 1 was specifically enriched in long-chain fatty acid metabolism and organic phosphate metabolism; Cluster 2 was enriched in morphogenesis regulation and mRNA binding, reflecting dual roles in organogenesis and post-transcriptional regulation; Cluster 3 was primarily enriched in broad membrane system components including membrane-bounded organelles; most notably, Cluster 4 was highly specifically enriched in phosphatidylinositol-3,4,5-trisphosphate 5-phosphatase, phosphatidylinositol-4,5-bisphosphate phosphatase, and trehalose phosphatase activities—functional categories entirely absent from WGD duplicate genes—indicating significant neofunctionalization of TRD duplicates through the phosphatidylinositol signaling pathway. Overall, WGD genes tend to maintain conserved co-expression associated with core cellular functions, whereas TRD genes undergo greater functional specialization and expression divergence. To evaluate the influence of the co-expression definition threshold on the above conclusions, we repeated the comparative analysis of co-expression rates using *r* = 0.4 and *r* = 0.6 as alternative cutoffs. The results showed that, under both thresholds, WGD gene pairs exhibited significantly higher co-expression rates than TRD gene pairs (Figure S3a,b).

### 3.2. Gene Length Is Negatively Correlated with Co-Expression

We first calculated Pearson correlation coefficients between gene pairs and their average gene lengths to investigate factors influencing the divergent co-expression of the two gene types. The analysis revealed a slight negative correlation trend between gene length and co-expression strength (Figure S1c,d).

**Figure 2:**
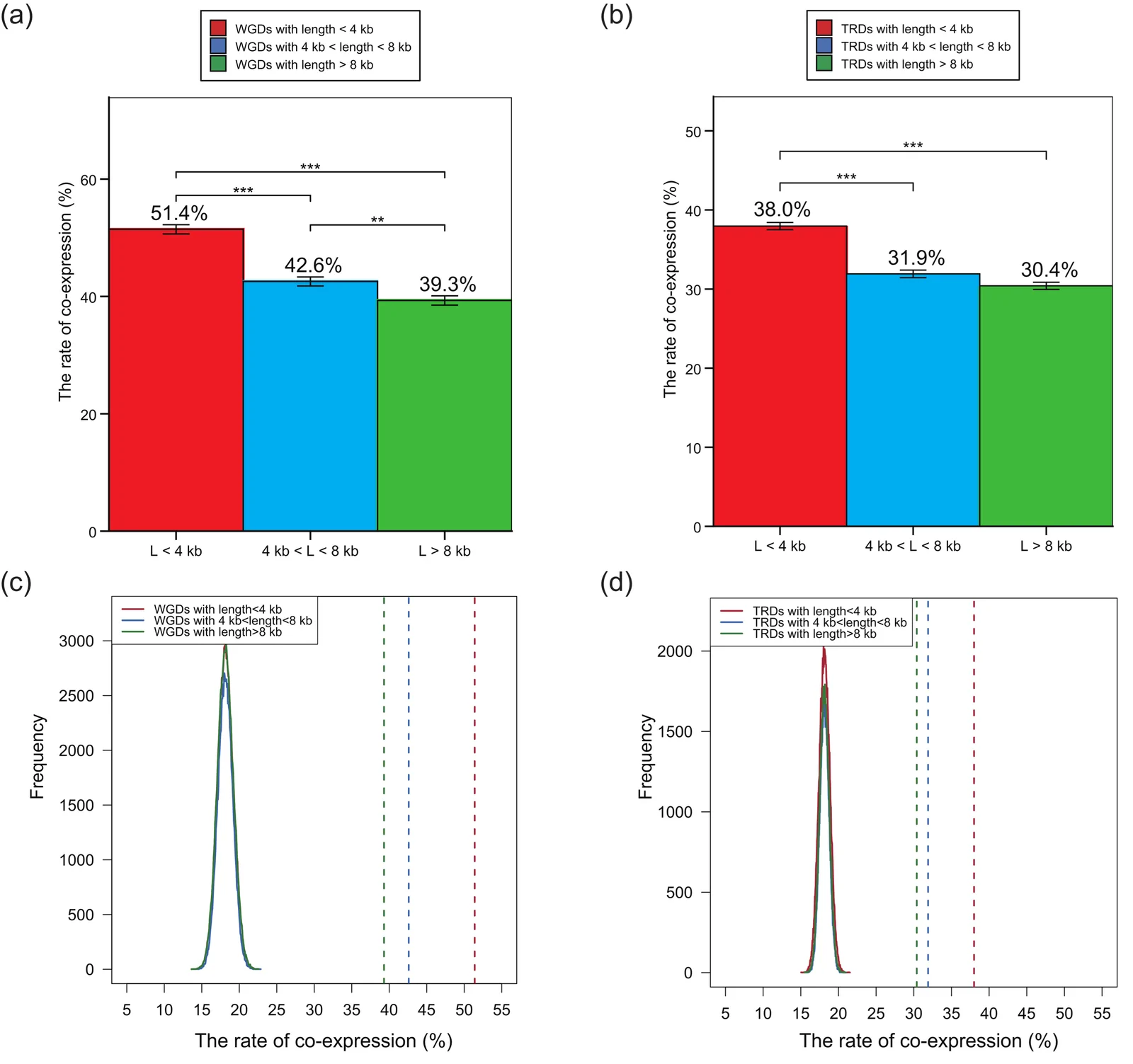
Effect of gene length on co-expression rates of WGD and TRD gene pairs. (**a**,**b**) Co-expression rate comparison of WGD and TRD gene pairs stratified by gene length groups, respectively. (**c**,**d**) Frequency distributions of co-expression ratios from 100,000 randomization experiments across three gene length ranges for WGD and TRD gene pairs, respectively. Each iteration sampled the same number of gene pairs as the empirical dataset. Error bars, bootstrapped standard errors. Significance values were determined by the Mann–Whitney U test (** *p* < 0.01, *** *p* < 0.001).

Length distribution analysis showed that TRD genes had a slightly higher distribution peak than WGD genes (Figure S2a), indicating that TRD genes tend to have longer gene bodies overall. To further evaluate the effect of gene length on co-expression patterns, all gene pairs were classified into three groups based on average length: short (≤4 kb; WGD: *n* = 1285, TRD: *n* = 2746), medium (4–8 kb; WGD: *n* = 1504, TRD: *n* = 3893), and long (≥8 kb; WGD: *n* = 1282, TRD: *n* = 3535). Co-expression rates were inversely related to gene length. Specifically, the co-expression rates of WGD gene pairs decreased progressively along the length gradient: short (51.4%) > medium (42.6%) > long (39.3%); TRD gene pairs showed a similar trend: short (38.0%) > medium (31.9%) > long (30.4%); these differences were statistically significant (Mann–Whitney U test, *p* < 0.05; Figure 2a,b). Notably, WGD co-expression rates exceeded TRD rates in all length categories. To determine whether these observations could be attributed to random factors, a randomization test was performed in which the same number of gene pairs were randomly sampled and co-expression rates calculated, repeated 100,000 times. Co-expression rate frequencies in the randomized groups were markedly lower than those observed empirically (Mann–Whitney U test, *p* < 2.2 × 10^−16^; Figure 2c,d), confirming that the length effect on co-expression represents a genuine biological phenomenon rather than sampling bias.

### 3.3. Sequence Similarity Is Negatively Correlated with Expression Divergence

**Figure 3:**
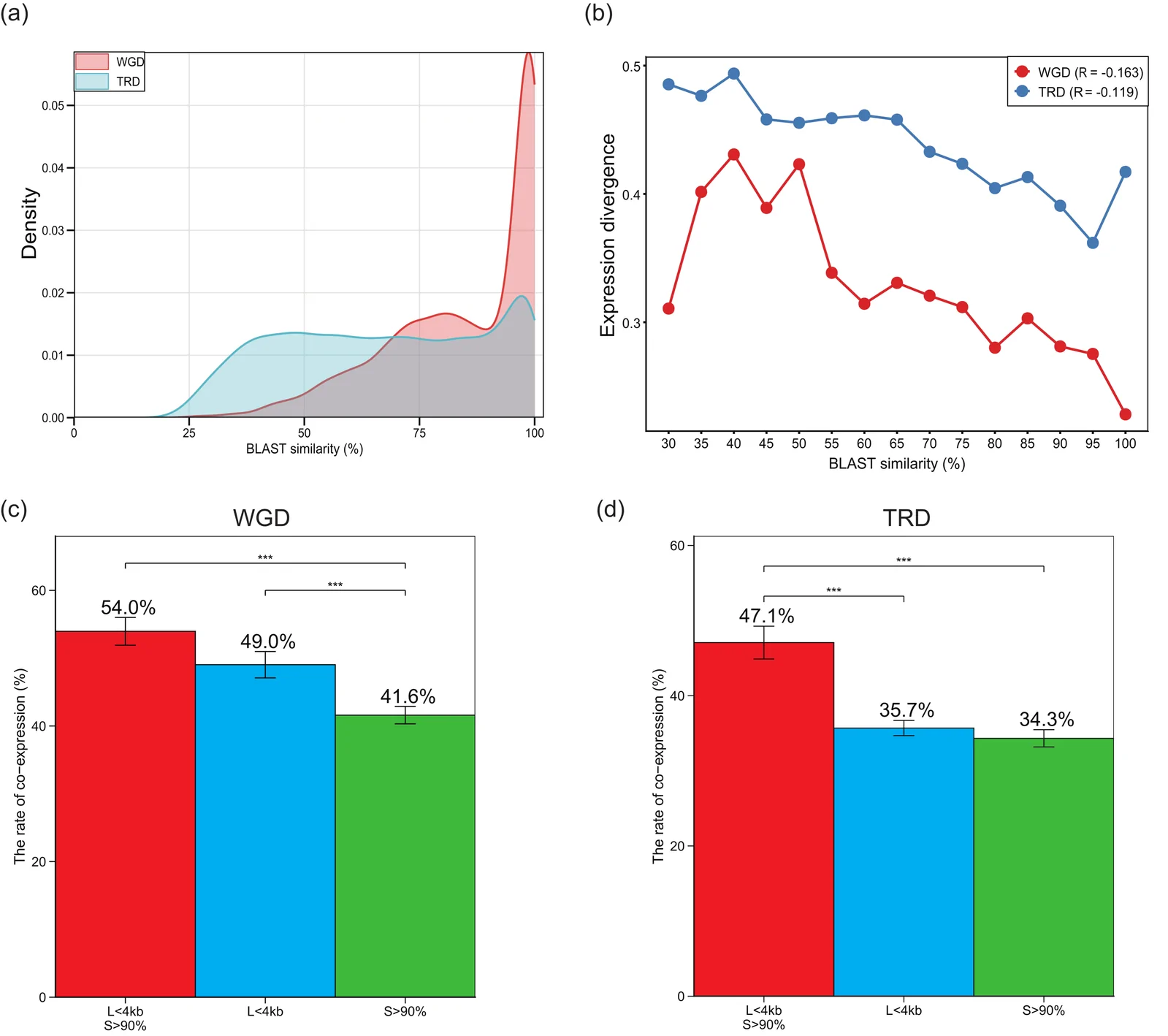
Synergistic effects of sequence similarity and gene length on co-expression of WGD and TRD gene pairs. (**a**) Kernel density distribution of BLAST sequence similarity for WGD and TRD gene pairs. (**b**) Association between BLAST sequence similarity and expression divergence. (**c**,**d**) Co-expression rates of WGD and TRD gene pairs, respectively, under conditions of high sequence similarity and short gene length. Error bars, bootstrapped standard errors. Significance values were determined by the Mann–Whitney U test (*** *p* < 0.001).

To elucidate the role of sequence similarity in regulating co-expression of gene pairs, we calculated BLAST (version 2.13.0)-based sequence identity percentages for all WGD and TRD gene pairs. Density distribution analysis revealed fundamentally different distribution patterns between the two gene types (Figure 3a). WGD gene pairs showed a relatively concentrated distribution in the 70–90% similarity range with a unimodal pattern, whereas TRD gene pairs exhibited a broader distribution (40–100%) with a relatively flat profile. Association analysis with expression divergence revealed a non-linear relationship across the entire similarity range (30–100%), with both gene types displaying a similar basic trend of slightly decreasing expression divergence as BLAST similarity increased (Figure 3b), suggesting that the overall regulatory effect of sequence similarity on expression divergence is limited.

To evaluate the effects of sequence similarity and gene length on the co-expression of WGD and TRD gene pairs, we first classified these gene pairs accordingly. Based on the bimodal distribution of genome-wide gene lengths, with a valley at approximately 4 kb for both duplication classes, genes were categorized as short (≤4 kb) or long (>4 kb). Meanwhile, following the criteria established by Giannuzzi et al. in the grapevine genome [33], and based on the clear inflection point at 90% in the similarity distribution of WGD gene pairs (Figure 3a), we adopted 90% as the threshold to divide gene pairs into high-similarity (>90%) and low-similarity (≤90%) groups.

The most biologically significant finding was that a remarkable synergistic enhancement of co-expression rates was observed for both gene classes when BLAST similarity exceeded 90% and gene length was below 4 kb (Figure 3c,d). Quantitative analysis showed that, among WGD gene pairs, those with high similarity (>90%) and short gene length (<4 kb) exhibited a co-expression rate of 54.0% (*n* = 673), representing a 12.4 percentage-point increase compared with all high-similarity WGD gene pairs (41.6%, *n* = 2020). Among TRD gene pairs, the corresponding group showed a co-expression rate of 47.1% (*n* = 1376), an 11 percentage-point increase relative to all high-similarity TRD gene pairs (34.3%, *n* = 2225). Most notably, the WGD group with both high similarity and short gene length (54.0%) significantly outperformed the corresponding TRD group (47.1%), with a difference of 7.0 percentage points (Mann–Whitney U test, *p* < 0.001). These results indicate that high sequence similarity and short gene length jointly favor co-expression, and this combined bias is more pronounced in WGD genes than in TRD genes. Taken together, these findings suggest a possible dual-constraint model governing co-expression maintenance: high sequence conservation ensures functional constraint—that is, both copies retain similar biochemical functions under comparable selective pressures—whereas shorter gene body length reduces the likelihood of transcriptional noise being introduced during transcription elongation. Both conditions appear to be necessary for the robust maintenance of co-expression.

### 3.4. ATAC-Seq and H3K27ac Peaks Enhance Transcriptional Expression and Co-Expression of Duplicate Genes

Given that non-coding intergenic regions are enriched with diverse transcriptional regulatory elements, genome-wide analysis of these elements is essential for understanding their effects on transcriptional activity. Publicly available ATAC-seq and H3K27ac ChIP-seq raw datasets from recent studies were processed (see Section 2). Peaks in both datasets were identified using MACS2, and only peaks reproducibly detected across biological replicates were retained (Figure S2b).

**Figure 4:**
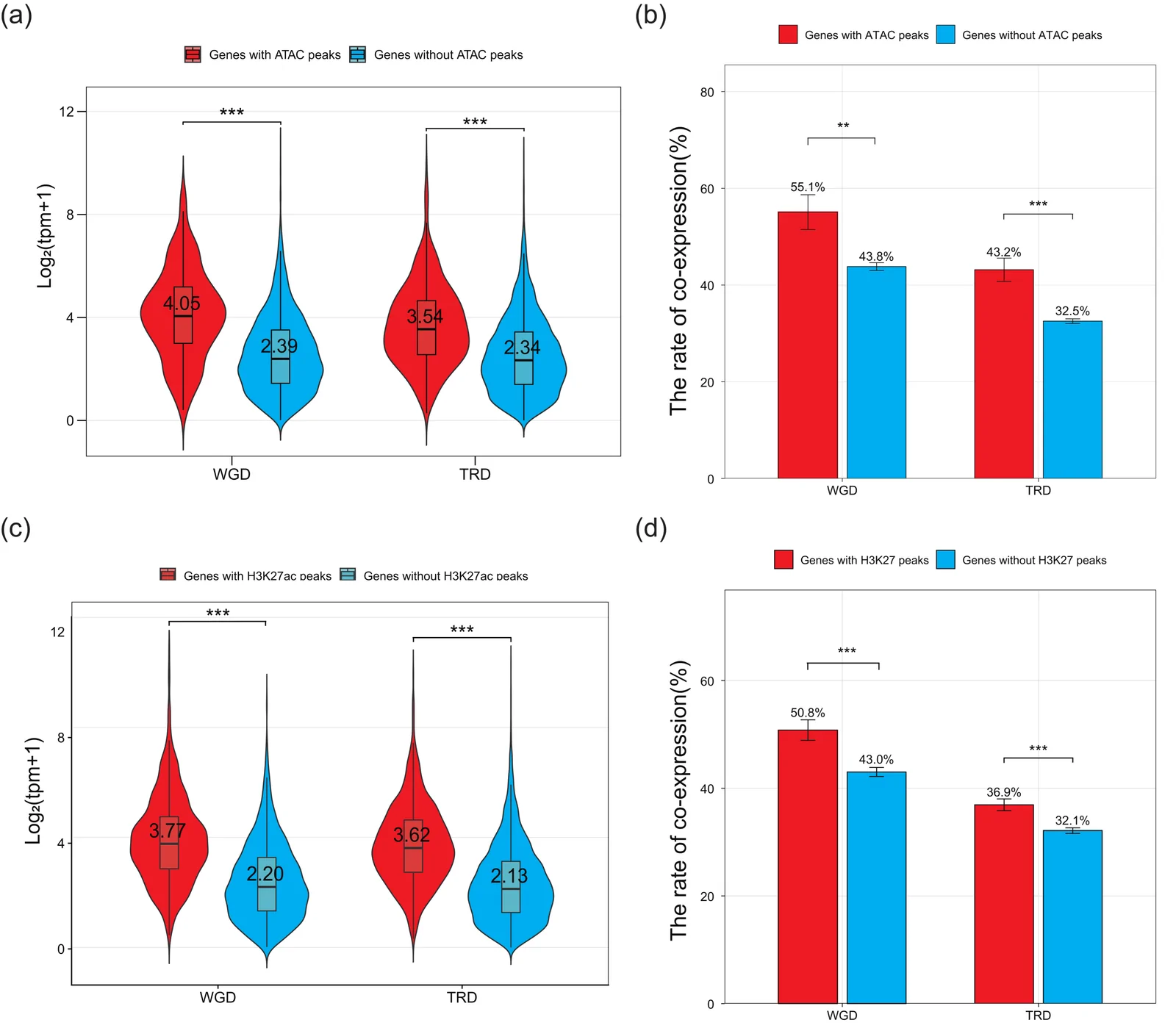
Chromatin accessibility and H3K27ac modification promote expression and co-expression of WGD and TRD gene pairs. (**a**,**c**) Expression levels of WGD and TRD genes with versus without ATAC-seq and H3K27ac peak annotation, respectively. (**b**,**d**) Co-expression rates of gene pairs stratified by ATAC-seq and H3K27ac peak annotation status, respectively. Error bars, bootstrapped standard errors. Significance values were determined by the Mann–Whitney U test (** *p* < 0.01, *** *p* < 0.001).

To investigate whether the presence of ATAC-seq or H3K27ac peaks correlates with elevated gene expression, both gene types were annotated with ATAC-seq and H3K27ac signals. WGD and TRD genes with ATAC peaks both showed significantly elevated expression levels. WGD genes with ATAC peaks displayed a median expression value of 4.05 [log_2_(TPM+1)], substantially higher than genes without peaks (2.39). Similarly, TRD genes with ATAC peaks showed a median expression of 3.54 versus 2.34 for those without peaks (Figure 4a). Critically, the presence of accessible chromatin not only enhanced gene expression but also increased co-expression rates between duplicate gene pairs. Among WGD gene pairs, those in which both genes contained ATAC peaks showed a co-expression rate of 55.1%, significantly higher than pairs without ATAC peaks (43.8%). The same pattern was observed in TRD genes, with ATAC peak-positive pairs reaching 43.2% co-expression versus 32.5% for ATAC peak-negative pairs (Mann–Whitney U test, *p* < 0.001; Figure 4b).

To further validate these findings, we examined H3K27ac modification. Consistent with ATAC-seq results, gene pairs with H3K27ac marks showed higher expression levels (WGD: 3.77 vs. 2.20; TRD: 3.62 vs. 2.13; Figure 4c) and higher co-expression rates (Figure 4d). These results indicate that open chromatin and active histone modifications are each positively associated with expression levels and co-expression rates of duplicate gene pairs, and that these associations are more pronounced in WGD genes than in TRD genes, suggesting that WGD genes may possess more complete cis-regulatory networks to maintain co-expression.

### 3.5. DNA Methylation Displays Distinct Patterns in WGD and TRD Genes

**Figure 5:**
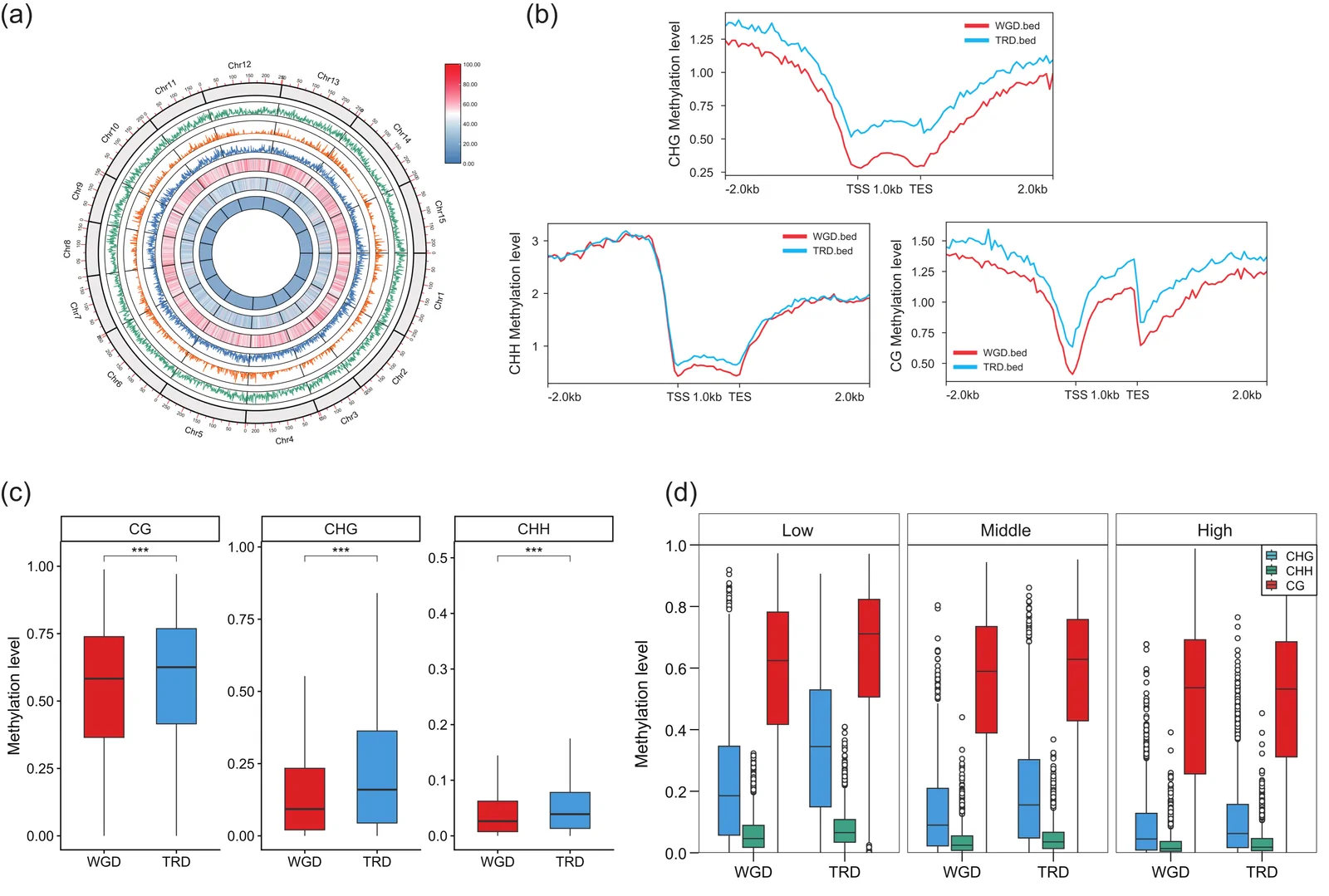
DNA methylation landscape of the tea plant genome. (**a**) Genome-wide distribution of CG, CHG, and CHH methylation across 15 chromosomes, with tracks for whole-genome density, WGD gene density, TRD gene density, and methylation levels per context (1 Mb windows). (**b**) Methylation profiles across gene bodies and 2 kb flanking regions of WGD and TRD genes in three sequence contexts. TSS, transcription start site; TTS, transcription termination site. (**c**) Differential DNA methylation levels between WGD and TRD genes. (**d**) Methylation levels of WGD and TRD genes stratified by expression level (low, medium, high) in three sequence contexts. Significance values were determined by the Mann–Whitney U test (*** *p* < 0.001).

To dissect the epigenetic basis of expression differences between WGD and TRD genes, we systematically analyzed three types of DNA methylation levels, gene density, and repetitive element density across all 15 chromosomes of the tea plant genome using 1 Mb windows. The genome-wide methylation map showed significantly elevated methylation in regions with high repetitive element density, whereas gene-enriched regions exhibited relatively lower methylation levels (Figure 5a). Chromosomal distribution analysis indicated that WGD genes tend to cluster in gene-dense regions of chromosome arms, while TRD genes are more dispersedly distributed.

Methylation comparisons across gene bodies and flanking regions revealed that TRD genes had higher methylation levels in all three contexts than WGD genes. Both gene types showed methylation troughs around the TSS and TTS with the lowest methylation in gene bodies (Figure 5b). Notably, in contrast to the pattern of CG and CHG methylation gradually decreasing from flanking regions toward the TSS, CHH methylation displayed the opposite trend—rising markedly from flanking regions toward the TSS, revealing a unique epigenetic feature of CHH islands in tea plant gene flanking regions. To our knowledge, this characteristic is rarely reported in plant genome methylation studies, suggesting that the elevated CHH methylation near gene boundaries may reflect RdDM-related targeting activity, warranting further investigation with small RNA and functional evidence.

To further characterize the epigenetic divergence between WGD and TRD genes, we compared their global DNA methylation profiles across three sequence contexts (CG, CHG, and CHH). TRD genes exhibited significantly higher methylation levels than WGD genes across all three methylation contexts (Figure 5c). Furthermore, stratified analysis dividing genes into low, medium, and high expression groups further revealed dynamic methylation regulatory patterns. In the low-expression group, TRD gene CG and CHG methylation were markedly higher than WGD; as expression levels increased, the inter-group differences in non-CG methylation progressively narrowed, while the differential repression of TRD genes by CG methylation persisted; in the high-expression group, CHG and CHH methylation converged, but TRD gene CG methylation remained at relatively elevated levels (Figure 5d). This suggests that CG methylation may represent a relatively stable epigenetic modification associated with TRD gene expression repression, and appears less responsive to transcriptional activation signals compared with non-CG methylation.

In summary, WGD genes reside in a relatively open chromatin environment with low levels of all three methylation modifications, which is associated with stable high-level transcription, whereas TRD genes show elevated CG, CHG, and CHH methylation. These patterns, together with the chromatin accessibility findings, highlight important epigenetic features associated with expression divergence between WGD and TRD genes in tea plants.

### 3.6. ACR Number Regulates Expression of WGD and TRD Genes

**Figure 6:**
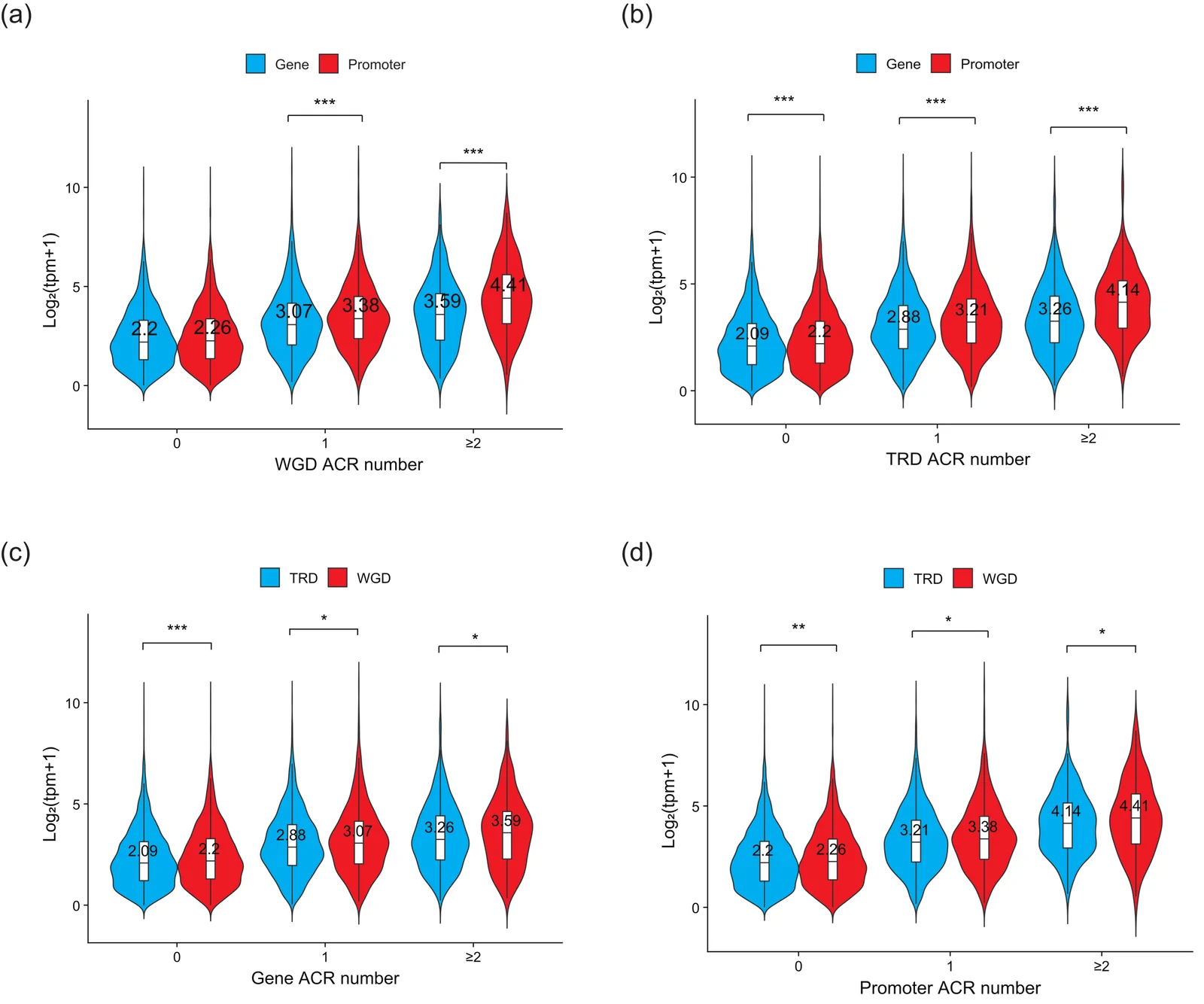
Dose-dependent regulation of gene expression by ACR number in WGD and TRD genes. (**a**,**b**) Expression level distributions of WGD and TRD genes across gene body ACR number categories, respectively. (**c**,**d**) Expression level comparisons between WGD and TRD genes within the same gene body and promoter ACR number categories, respectively. Significance values were determined by the Mann–Whitney U test (* *p* < 0.05, ** *p* < 0.01, *** *p* < 0.001).

To investigate the differential effects of accessible chromatin region (ACR) number on expression regulation of WGD and TRD genes, we systematically analyzed the correlation between ACR number in gene bodies and promoter regions and gene expression levels. Using log_2_(TPM+1) as the expression metric, expression levels in both WGD and TRD genes increased significantly with increasing ACR number in gene bodies and promoters (Figure 6a,b), consistent with the classical regulatory model that open chromatin facilitates transcription factor binding and drives gene expression. Further comparison of expression differences between WGD and TRD genes under equivalent ACR numbers revealed significantly different regulatory patterns. When gene body ACR number was 0, TRD gene expression (2.09) was significantly lower than WGD gene expression (2.22); when gene body ACR number increased to 1 or 2, TRD gene expression remained significantly lower than WGD, suggesting that WGD genes exhibit higher basal transcriptional activity under equivalent chromatin accessibility. Similar patterns were observed in promoter ACR analyses (Mann–Whitney U test, *p* < 0.001; Figure 6c,d). Under equivalent chromatin accessibility conditions, TRD genes still exhibited significantly lower expression levels than WGD genes, with persistent CG methylation representing one potential factor contributing to this difference, suggesting that even when chromatin accessibility converges, CG methylation may remain involved in maintaining transcriptional repression of TRD genes.

### 3.7. Differential Effects of SNP Variation on WGD and TRD Gene Expression and Co-Expression

**Figure 7:**
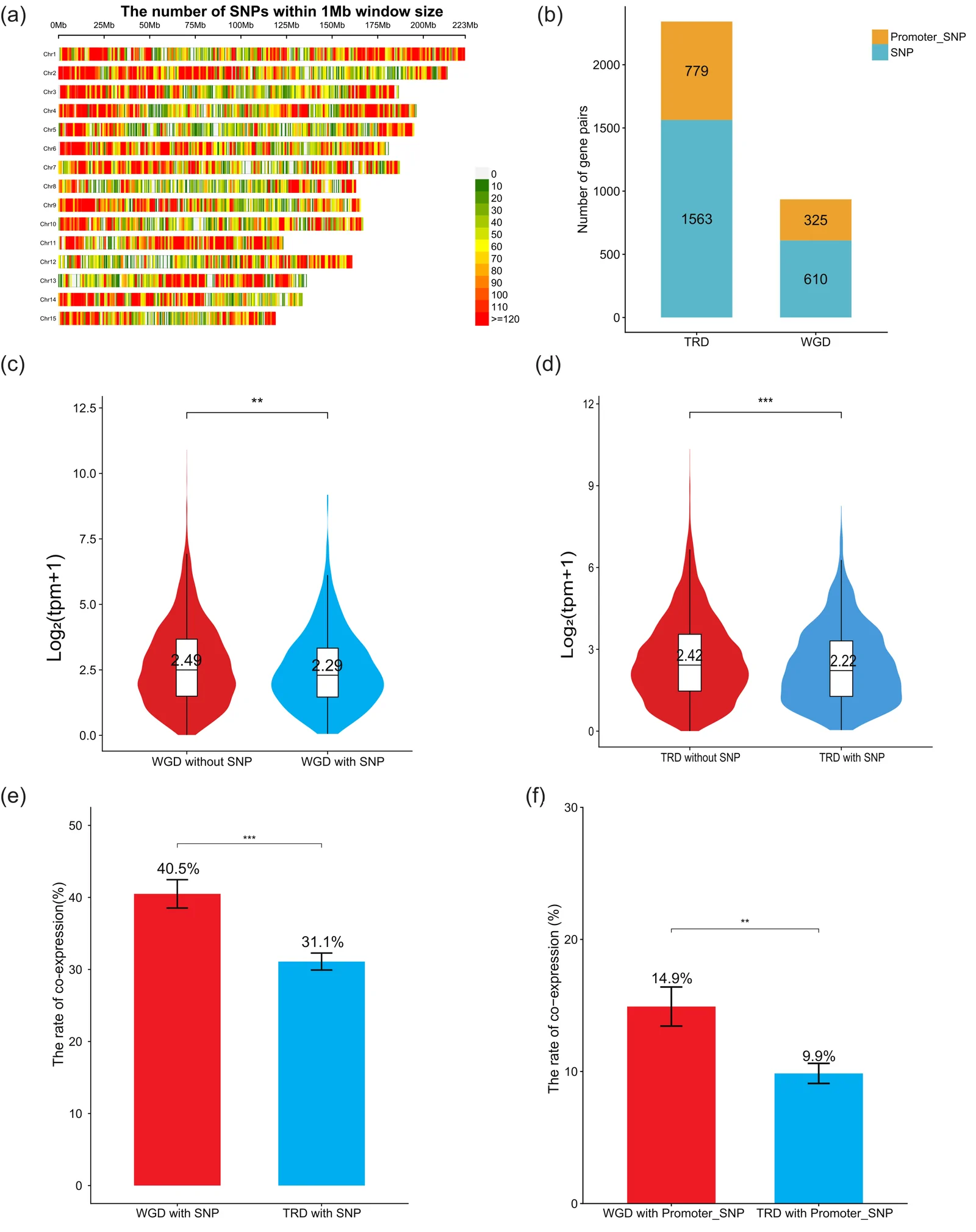
Differential effects of SNP variation on expression and co-expression of WGD and TRD genes. (**a**) Genome-wide SNP density heatmap across 15 chromosomes. (**b**) Gene body SNP only (SNPs located exclusively in gene body regions) and promoter SNP only (SNPs located exclusively in promoter regions) counts in WGD and TRD gene pairs. (**c**,**d**) Expression levels of WGD and TRD genes with versus without SNP loci, respectively. (**e**,**f**) Co-expression rates of WGD and TRD gene pairs harboring gene body and promoter-region SNPs, respectively. Error bars, bootstrapped standard errors. Significance values were determined by the Mann–Whitney U test (** *p* < 0.01, *** *p* < 0.001).

To systematically evaluate the contribution of SNP variation to regulatory divergence between WGD and TRD genes, we conducted a comprehensive analysis of genome-wide SNP distribution patterns and their effects on gene expression and co-expression. Heatmap visualization showed significant regional heterogeneity in SNP distribution across the 15 tea plant chromosomes, with high-density regions primarily concentrated in the mid-arms and some terminal regions of chromosomes, while pericentromeric regions showed relatively low density (Figure 7a), consistent with the general pattern of low recombination rates and high sequence conservation in plant genome centromeric regions.

Gene pairs were categorized into gene body SNP-only and promoter SNP-only groups (Figure 7b). Expression level comparisons revealed that in both WGD and TRD genes, the median expression values of the no SNP groups (WGD: 2.49; TRD: 2.42) were significantly higher than those of gene body SNP only groups (WGD: 2.29; TRD: 2.22), with the repressive effect of gene body SNP only on expression significantly stronger in TRD genes than WGD genes (Figure 7c,d). In co-expression rate analyses, among all gene pairs containing gene body SNP only, WGD gene pairs maintained a co-expression rate (40.5%) significantly higher than TRD (31.1%), with a difference of 9.4 percentage points (Figure 7e). These findings indicate that WGD gene pairs maintain a higher degree of coordinated expression even in the presence of reported SNP loci. Among gene pairs carrying promoter SNPs, the co-expression rates of both WGD (14.9%) and TRD (9.9%) dropped substantially compared with those of gene pairs with all types of SNPs (Figure 7f), suggesting that promoter-localized SNP loci are closely associated with reduced co-expression. Taken together, the presence of reported SNP loci—particularly those located in promoter regions—is significantly associated with duplicate gene expression divergence and co-expression network remodeling, with SNP-carrying genes showing lower median expression levels in both duplication types.

### 3.8. Weighted Gene Co-Expression Network Analysis Reveals Tissue-Specific Modules

**Figure 8:**
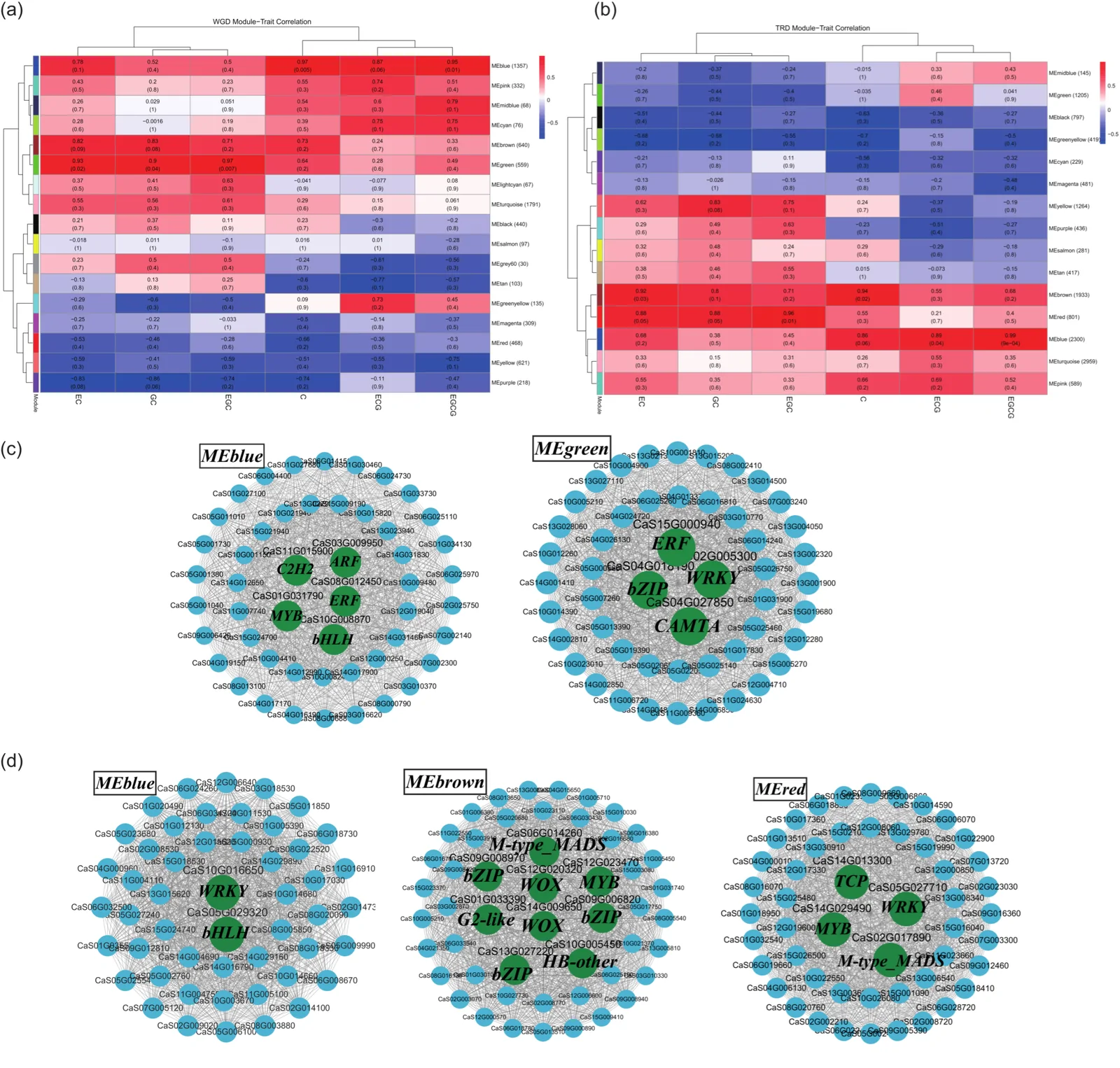
WGCNA-based co-expression network analysis and catechin-associated transcription factor networks of WGD and TRD genes. (**a**,**b**) Module–trait correlation heatmaps between WGD and TRD WGCNA modules and six catechin metabolite accumulation levels, respectively. Pearson *r* and *p*-values are shown at intersections; parenthetical numbers indicate gene counts per module. (**c**,**d**) Network visualizations of hub genes from key WGD and TRD modules, respectively, with predicted transcription factors (TFs) highlighted.

Weighted gene co-expression network analysis (WGCNA) is a highly robust method for classifying genes through hierarchical clustering of gene co-expression networks. To investigate the functional contributions of both duplicate gene types to catechin metabolic regulation, we constructed separate weighted co-expression networks for WGD and TRD genes and correlated module eigengenes with catechin accumulation levels (Figure S2c,d). A total of 17 modules were identified from WGD genes, of which two were highly correlated with metabolites (*r* ≥ 0.8, *p* < 0.05). Specifically, the MEblue module contained 1357 genes and was highly significantly positively correlated with epigallocatechin gallate (EGCG) and catechin (C). The MEgreen module contained 559 genes (7.6%) and was significantly positively correlated with epigallocatechin (EGC), epicatechin (EC), and gallocatechin (GC) (Figure 8b, left). WGCNA of TRD genes identified 15 modules, of which three were highly correlated with metabolites (*r* ≥ 0.8, *p* < 0.05). The MEbrown module contained 1933 genes (13.5%) and was significantly positively correlated with epicatechin (EC) and catechin (C). The MEred module contained 801 genes (5.6%) and was highly significantly positively correlated with epigallocatechin (EGC). The MEblue module contained 2300 genes (16.1%) and was highly significantly positively correlated with both EGCG and epicatechin gallate (ECG) (Figure 8b, right), suggesting that these gene networks share similar regulatory features involved in catechin biosynthesis.

Hub genes within modules are generally considered to represent the biological function of those modules. We therefore constructed co-expression networks based on the 50 genes with the highest module membership values (KME, connectivity based on key module eigengenes) in each of the five modules, with transcription factors (TFs) selected as key hub genes. Five TFs were identified in the WGD MEblue module: C2H2, ARF, ERF, MYB, and bHLH; four TFs were identified in the MEgreen module: ERF, bZIP, WRKY, and CAMTA (Figure 8c). These TFs are highly co-expressed with catechin accumulation modules, suggesting they may serve as candidate regulatory factors in catechin biosynthesis, though their specific regulatory relationships await functional experimental validation. The TRD hub gene network revealed that the top 50 core genes of the MEblue module (associated with EGCG and ECG accumulation) contained WRKY and bHLH transcription factors, suggesting their potential involvement in the regulation of esterified catechin accumulation. In contrast, the core genes of the MEbrown module (associated with EC and C accumulation) included M-type MADS-box, WOX, bZIP, and MYB transcription factors (Figure 8d). Most notably, M-type MADS-box transcription factors have previously been reported primarily in floral organ development and endosperm function; their potential role in catechin biosynthesis regulation has not been systematically reported and represents one of the most exploration-worthy novel findings of this study, awaiting validation through EMSA, yeast one-hybrid, and genetic transformation approaches. Taken together, WGD key modules were primarily associated with simple catechin (EC, GC, EGC) accumulation, while one TRD module showed stronger associations with esterified catechins (ECG, EGCG), revealing the evolutionary mechanism by which WGD and TRD duplicate genes cooperatively drive diversified catechin accumulation through differential transcriptional regulatory networks.

## 4. Discussion

### 4.1. Do the Regulatory Factors Underlying Divergent Co-Expression Directly Drive Functional Differentiation of WGD and TRD Genes?

A deeper question underlying our multi-layered findings is whether the structural, epigenetic, and genetic regulatory differences identified here simultaneously drive functional divergence between WGD and TRD gene pairs. Converging lines of evidence suggest these two processes are intimately linked. By integrating transcriptomic and methylomic datasets, Chen et al. directly demonstrated that CG and CHH methylation levels at CsLAR and CsSCPL1A loci are highly concordant with stage-specific transcript abundance across leaf developmental stages and significantly correlated with the accumulation of corresponding catechin monomers [34], indicating that methylation-driven expression divergence directly shapes the partitioning of metabolic flux through the catechin biosynthetic pathway. From an evolutionary perspective, WGD gene pairs are subject to strong dosage-balance constraints that favor expression conservation and functional redundancy, whereas TRD copies, having been transposed to novel genomic loci, disengage from their ancestral *cis*-regulatory landscapes and thereby acquire the structural prerequisite for accelerated functional divergence [35]. Studies of WGD duplicate gene pairs in soybean further demonstrate that gain or loss of flanking regulatory sequences constitutes the primary driver of transcriptional divergence [36], and recent analyses of allotetraploid cotton confirm that subgenome asymmetries in chromatin accessibility directly determine homeolog expression bias, establishing chromatin structural dynamics as a central determinant of *cis*-regulatory landscape evolution and duplicate gene expression divergence in polyploids [37]. Future experiments employing CRISPR-dCas9-mediated targeted demethylation at the promoter of the CsLAR-TRD copy will provide a direct causal test of whether methylation-driven expression suppression is sufficient to drive functional fate transition.

### 4.2. Putative Mechanisms Linking TRD Module Transcription Factors to Catechin Traits

WGCNA identified two TRD modules significantly associated with catechin traits. The MEblue module was highly correlated with the accumulation of esterified catechins (EGCG and ECG) and predominantly involved WRKY and bHLH transcription factors, whereas the MEbrown module was associated with EC and C accumulation and contained M-type MADS-box, WOX, bZIP, and MYB transcription factors. WRKY and bHLH transcription factors have been previously implicated in the regulation of flavonoid and catechin biosynthesis in plants [38,39], suggesting that the WRKY and bHLH factors involved in the MEblue module may participate in catechin-related regulatory networks. In tea plants, the biosynthesis of EGCG and ECG from catechin precursors depends on the CsSCPL4/CsSCPL5-mediated galloylation reaction [40,41]. We therefore speculate that WRKY and bHLH factors in the MEblue module may be involved in these regulatory networks by binding to the promoters of SCPL1A-clade genes or by forming complexes with CsWRKY57like. The M-type MADS-box and WOX transcription factors identified in the MEbrown module were associated with EC and C accumulation. M-type MADS-domain proteins have rarely been reported in plant secondary metabolism [42]; however, HbMADS4 has been shown to negatively regulate HbSRPP in Hevea brasiliensis [43], and MIKC*-type MADS-box genes have been implicated in the regulation of carotenoid accumulation [44], suggesting that the functional repertoire of the MADS family in specialized metabolism warrants further investigation. WOX transcription factors are primarily known for their roles in meristem maintenance and stem cell regulation, with no previous reports linking them to secondary metabolism. Thus, the presence of M-type MADS-box and WOX factors in the MEbrown module provides new candidate gene resources for simple catechin regulation, although their functions require experimental validation. Meanwhile, the MYB-bHLH regulatory network involved in simple catechin biosynthesis in WGD modules has been supported by molecular evidence [39], providing independent support for the module-level functional assignments in this study. All associations between the above transcription factors and catechin traits are based on co-expression analyses and require further experimental validation through yeast one-hybrid assays, electrophoretic mobility shift assays (EMSA), and chromatin immunoprecipitation sequencing (ChIP-seq).

### 4.3. Future Directions

Two major limitations constrain the current analytical framework while also pointing to the most valuable directions for future investigation. First, the causal relationships among regulatory layers remain unresolved. The hierarchical order among promoter SNPs, CG methylation and chromatin accessibility—specifically, whether SNP-mediated disruption of methyltransferase recognition sequences precedes and promotes the establishment of CG methylation, or vice versa—cannot be determined from correlative multi-omics data alone. Allele-specific expression (ASE) analysis and CRISPR–dCas9 epigenome editing represent the most direct strategies for resolving these causal relationships. The seasonal oscillation of global methylome levels in tea shoots, which synchronizes with the dynamics of catechin accumulation, provides a natural system for ASE analysis across developmental time points [45]. Furthermore, the observation that newly formed and evolved autopolyploid Arabidopsis arenosa retains differential chromatin accessibility following whole-genome duplication supports the incorporation of time-resolved ATAC-seq into future experimental designs [46]. Second, the use of a single cultivar limits the generalizability of our conclusions. This study was conducted exclusively with the YK10 cultivar, and it remains unclear whether the hypermethylation patterns of TRD genes identified here are conserved across tea germplasm or are cultivar-specific. Comparative methylome analyses across publicly available cultivar genome assemblies (Shuchazao, Longjing 43, Tieguanyin, etc.) would enable the systematic identification of cultivar-specific differentially methylated regions within TRD promoter-associated ACRs, which represent high-priority targets for precision breeding aimed at EGCG enrichment. Combining such genomic targeting with CRISPRa-mediated transcriptional activation offers a transgene-free strategy for finely modulating the ratio of esterified to non-esterified catechins [47]. Additionally, in discussing the differential effects of WGD versus TRD on gene family evolution, it is important to acknowledge that our classification of genes primarily by duplication mechanism does not fully account for confounding factors such as duplication age (Ks) and transposable element (TE) proximity. WGD and TRD events may have occurred at markedly different times, and the distribution of TEs in the genome can influence the epigenetic regulation and expression patterns of neighbouring genes. These differences could confound the effects attributable to the duplication mechanism itself [48,49].

## 5. Conclusions

Gene duplication provides raw material for functional innovation, yet the regulatory mechanisms governing whether duplicate genes undergo expression conservation or divergence—and the resultant divergent co-expression patterns—remain elusive in polyploid crops. Using tea plant (*C. sinensis*) as a model, this study constructed a multi-layered framework integrating structural constraints, epigenetic regulation, and transcription factor networks to investigate transcriptional divergence between WGD and TRD duplicate genes, and directly linked this framework to the diversified accumulation of catechin metabolites.

At the structural level, gene length and sequence similarity jointly favor co-expression of duplicate genes: shorter gene body length is associated with higher co-expression, whereas lower sequence similarity correlates with greater expression divergence. These factors act synergistically rather than independently to maintain duplicate gene co-expression, offering new perspectives on the structural determinants of duplicate gene retention and divergence.

At the epigenetic level, the dynamic balance between activating and repressive signals determines the transcriptional fate of duplicate genes. Chromatin accessibility (ATAC-seq signals) and H3K27ac modification are each associated with enhanced transcription and co-expression of duplicate genes. TRD genes are subject to multi-layered repressive regulation: elevated CG methylation in promoter regions contributes to transcriptional repression, while promoter SNPs further exacerbate expression divergence of TRD genes by disrupting cis-regulatory element integrity. These results indicate that WGD and TRD genes reside in distinct epigenetic landscapes—the former dominated by activating signals and the latter additionally constrained by methylation and genetic variation barriers.

At the functional level, by integrating co-expression networks with metabolite accumulation data, we identified multiple key transcription factors involved in catechin biosynthesis and revealed functional partitioning among duplicate genes: WGD modules showed significant associations with EC, GC, and EGC accumulation, whereas the TRD MEblue module exhibited strong associations with EGCG and ECG. The module–trait relationships of the two gene classes displayed both divergence and overlap, collectively driving the diversification of catechin components in tea plant through differentiated transcriptional regulation.

In summary, this study proposes a duplicate gene expression divergence model integrating structural, epigenetic, and transcriptional regulation, and systematically elucidates the mechanisms underlying metabolic diversity in the ancient polyploid genome of tea plant. This framework not only deepens our understanding of duplicate gene evolutionary dynamics in plants but also provides concrete molecular targets for precision breeding centered on EGCG, including SNP sites within accessible chromatin regions (ACRs) of promoters, differentially methylated regions (DMRs), and key transcription factor nodes such as MADS-box and bZIP. These candidate targets offer a theoretical foundation and actionable entry points for future integrated applications based on epigenome editing (e.g., CRISPRa/dCas9) and molecular-assisted breeding.

## Data Availability

All data used in this study were obtained from public databases. The high-quality reference genome of *Camellia sinensis* cultivar YK10 is available from TeaBase [50]. RNA-seq datasets were generated from the YK10 cultivar and are deposited in the NCBI Sequence Read Archive (SRA) under accession numbers PRJNA381277 and PRJCA574928 [51,52]. SNP data for YK10 were downloaded from TeaGVD (http://www.teaplant.top/teagvd (accessed on 24 July 2026)) [53]. All other multi-omics datasets, including ATAC-seq, H3K27ac ChIP-seq, and whole-genome bisulfite sequencing (WGBS), were generated from the *C. sinensis* cultivar Tieguanyin and are available from the National Genomics Data Center (NGDC) under project number PRJCA017759 (ATAC-seq and H3K27ac ChIP-seq) and PRJCA014523 (WGBS) [16]. Catechin accumulation data were extracted from the corresponding publications as detailed in Table S3. Detailed sample information, including cultivar origin, tissue type, developmental stage, and sequencing platform for each dataset, is provided in Table S3.

## Supplementary Materials

The following supporting information can be downloaded at https://www.mdpi.com/article/10.3390/genes17080898/s1. Figure S1: (a) Proportion of gene pairs with expression divergence in WGD and TRD genes across eight tissues. (b) Proportion of co-expressed gene pairs in WGD and TRD genes across four clusters. (c) Scatter plot showing the relationship between WGD average gene length and Pearson correlation coefficient. (d) Scatter plot showing the relationship between TRD average gene length and Pearson correlation coefficient. Figure S2: (a) Sequence similarity density distribution plot. (b) Bar chart showing ATAC and H3K27ac peak numbers. (c) Correlation plot of WGD module eigengenes with catechin accumulation levels. (d) Correlation plot of TRD module eigengenes with catechin accumulation levels. Figure S3: (a) Co-expression rates of WGD versus TRD gene pairs at the r > 0.4 threshold. (b) Co-expression rates of WGD versus TRD gene pairs at the r > 0.6 threshold. Table S1: Gene IDs of WGD gene pairs. Table S2: Gene IDs of TRD gene pairs. Table S3: Sources of transcriptomic and epigenomic data used in this study. Table S4: Summary of predicted transcription factors.

## Abbreviations

WGD	Whole-genome duplication
TRD	transposed duplication
GC	gallocatechin
EGCG	epigallocatechin gallate
ECG	epicatechin gallate
EGC	epigallocatechin
EC	epicatechin
C	catechin
KME	key module eigengene-based connectivity

## References

[1] Panchy N., Lehti-Shiu M., Shiu S.H. (2016). Evolution of Gene Duplication in Plants. Plant Physiol..

[2] Flagel L.E., Wendel J.F. (2009). Gene duplication and evolutionary novelty in plants. New Phytol..

[3] Jiao Y., Wickett N.J., Ayyampalayam S., Chanderbali A.S., Landherr L., Ralph P.E., Tomsho L.P., Hu Y., Liang H., Soltis P.S. (2011). Ancestral polyploidy in seed plants and angiosperms. Nature.

[4] Soltis D.E., Albert V.A., Leebens-Mack J., Bell C.D., Paterson A.H., Zheng C., Sankoff D., De Pamphilis C.W., Wall P.K., Soltis P.S. (2009). Polyploidy and angiosperm diversification. Am. J. Bot..

[5] Jiang N., Bao Z., Zhang X., Eddy S.R., Wessler S.R. (2004). Pack-MULE transposable elements mediate gene evolution in plants. Nature.

[6] Wang Y., Wang X., Tang H., Tan X., Ficklin S.P., Feltus F.A., Paterson A.H. (2011). Modes of gene duplication contribute differently to genetic novelty and redundancy, but show parallels across divergent angiosperms. PLoS ONE.

[7] Birchler J.A., Veitia R.A. (2012). Gene balance hypothesis: Connecting issues of dosage sensitivity across biological disciplines. Proc. Natl. Acad. Sci. USA.

[8] Ganko E.W., Meyers B.C., Vision T.J. (2007). Divergence in expression between duplicated genes in Arabidopsis. Mol. Biol. Evol..

[9] Wang Y., Tan X., Paterson A.H. (2013). Different patterns of gene structure divergence following gene duplication in Arabidopsis. BMC Genom..

[10] Yang C.S., Hong J. (2013). Prevention of chronic diseases by tea: Possible mechanisms and human relevance. Annu. Rev. Nutr..

[11] Chen D., Chen G., Sun Y., Zeng X., Ye H. (2020). Physiological genetics, chemical composition, health benefits and toxicology of tea (*Camellia sinensis* L.) flower: A review. Food Res. Int..

[12] Qu Z., Liu A., Li P., Liu C., Xiao W., Huang J., Liu Z., Zhang S. (2021). Advances in physiological functions and mechanisms of (-)-epicatechin. Crit. Rev. Food Sci. Nutr..

[13] Xia E., Tong W., Hou Y., An Y., Chen L., Wu Q., Liu Y., Yu J., Li F., Li R. (2020). The reference genome of tea plant and resequencing of 81 diverse accessions provide insights into its genome evolution and adaptation. Mol. Plant.

[14] Wei C., Yang H., Wang S., Zhao J., Liu C., Gao L., Xia E., Lu Y., Tai Y., She G. (2018). Draft genome sequence of *Camellia sinensis* var. sinensis provides insights into the evolution of the tea genome and tea quality. Proc. Natl. Acad. Sci. USA.

[15] Ye T., Li S., Li Y., Xiao S., Yuan D. (2024). Impact of polyploidization on genome evolution and phenotypic diversity in oil-tea Camellia. Ind. Crops Prod..

[16] Kong W., Yu J., Yang J., Zhang Y., Zhang X. (2023). The high-resolution three-dimensional (3D) chromatin map of the tea plant (*Camellia sinensis*). Hortic. Res..

[17] Buenrostro J., Giresi P., Zaba L., Chang H.Y., Greenleaf W.J. (2013). Transposition of native chromatin for fast and sensitive epigenomic profiling of open chromatin, DNA-binding proteins and nucleosome position. Nat. Methods.

[18] Creyghton M.P., Cheng A.W., Welstead G.G., Kooistra T., Carey B.W., Steine E.J., Hanna J., Lodato M.A., Frampton G.M., Sharp P.A. (2010). Histone H3K27ac separates active from poised enhancers and predicts developmental state. Proc. Natl. Acad. Sci. USA.

[19] Law J., Jacobsen S. (2010). Establishing, maintaining and modifying DNA methylation patterns in plants and animals. Nat. Rev. Genet..

[20] Qiao X., Li Q., Yin H., Qi K., Li L., Wang R., Zhang S., Paterson A.H. (2019). Gene duplication and evolution in recurring polyploidization-diploidization cycles in plants. Genome Biol..

[21] Chen S., Zhou Y., Chen Y., Gu J. (2018). Fastp: An ultra-fast all-in-one FASTQ preprocessor. Bioinformatics.

[22] Kim D., Langmead B., Salzberg S.L. (2015). HISAT: A fast spliced aligner with low memory requirements. Nat. Methods.

[23] Liao Y., Smyth G.K., Shi W. (2014). Feature Counts: An efficient general purpose program for assigning sequence reads to genomic features. Bioinformatics.

[24] Langmead B., Salzberg S.L. (2012). Fast gapped-read alignment with Bowtie 2. Nat. Methods.

[25] Danecek P., Bonfield J.K., Liddle J., Marshall J., Ohan V., Pollard M.O., Whitwham A., Keane T., McCarthy S.A., Davies R.M. (2021). Twelve years of SAMtools and BCFtools. GigaScience.

[26] Zhang Y., Liu T., Meyer C.A., Eeckhoute J., Johnson D.S., Bernstein B.E., Nusbaum C., Myers R.M., Brown M., Li W. (2008). Model-based analysis of ChIP-Seq (MACS). Genome Biol..

[27] Quinlan A.R., Hall I.M. (2010). BEDTools: A flexible suite of utilities for comparing genomic features. Bioinformatics.

[28] Zhou Q., Lim J.Q., Sung W.K., Li G. (2019). An integrated package for bisulfite DNA methylation data analysis with Indel-sensitive mapping. BMC Bioinform..

[29] Lescot M., Dehais P., Thijs G., Marchal K., Moreau Y., Van de Peer Y., Rouzé P., Rombauts S. (2002). PlantCARE, a database of plant cis-acting regulatory elements and a portal to tools for in silico analysis of promoter sequences. Nucleic Acids Res..

[30] Jin J., Tian F., Yang D.C., Meng Y.-Q., Kong L., Luo J., Gao G. (2017). PlantTFDB 4.0: Toward a central hub for transcription factors and regulatory interactions in plants. Nucleic Acids Res..

[31] Cantalapiedra C.P., Hernandez-Plaza A., Letunic I., Bork P., Huerta-Cepas J. (2021). EggNOG-mapper v2: Functional annotation, orthology assignments, and domain prediction at the metagenomic scale. Mol. Biol. Evol..

[32] Wu W., Lu M., Peng J., Lv H., Shi J., Zhang S., Liu Z., Duan J., Chen D., Dai W. (2022). Nontargeted and targeted metabolomics analysis provides novel insight into nonvolatile metabolites in Jianghua Kucha tea germplasm (*Camellia sinensis* var. *Assamica* cv. Jianghua). Food Chem. X.

[33] Giannuzzi G., D’Addabbo P., Gasparro M., Martinelli M., Carelli F.N., Antonacci D., Ventura M. (2011). Analysis of high-identity segmental duplications in the grapevine genome. BMC Genom..

[34] Chen J., Hu Y., Zhu Z., Zheng P., Liu S., Sun B. (2025). Dynamic DNA methylation modification in catechins and terpenoids biosynthesis during tea plant leaf development. Hortic. Plant J..

[35] Kaessmann H. (2010). Origins, evolution, and phenotypic impact of new genes. Genome Res..

[36] Fang C., Yang M., Tang Y., Zhang L., Zhao H., Ni H., Chen Q., Meng F., Jiang J. (2023). Dynamics of cis-regulatory sequences and transcriptional divergence of duplicated genes in soybean. Proc. Natl. Acad. Sci. USA.

[37] Hu G., Grover C.E., Vera D.L., Lung P.Y., Girimurugan S.B., Miller E.R., Conover J.L., Ou S., Xiong X., Zhu D. (2024). Evolutionary Dynamics of Chromatin Structure and Duplicate Gene Expression in Diploid and Allopolyploid Cotton. Mol. Biol. Evol..

[38] Luo Y., Huang X.X., Song X.F., Wen B.B., Xie N.C., Wang K.B., Huang J.A., Liu Z.H. (2022). Identification of a WRKY transcriptional activator from *Camellia sinensis* that regulates methylated EGCG biosynthesis. Hortic. Res..

[39] Wang W.L., Wang Y.X., Li H., Liu Z.W., Cui X., Zhuang J. (2019). Correction to: Two MYB transcription factors (CsMYB2 and CsMYB26) are involved in flavonoid biosynthesis in tea plant [*Camellia sinensis* (L.) O. Kuntze]. BMC Plant Biol..

[40] Yao S., Liu Y., Zhuang J., Zhao Y., Dai X., Jiang C., Wang Z., Jiang X., Zhang S., Qian Y. (2022). Insights into acylation mechanisms: Co-expression of serine carboxypeptidase-like acyltransferases and their non-catalytic companion paralogs. Plant J. Cell Mol. Biol..

[41] Chen X., Zhang X., Zhao Y., Gao L., Wang Z., Su Y., Zhang L., Xia T., Liu Y. (2024). Deactivating mutations in the catalytic site of a companion serine carboxypeptidase-like acyltransferase enhance catechin galloylation in Camellia plants. Hortic. Res..

[42] Masiero S., Colombo L., Grini P.E., Schnittger A., Kater M.M. (2011). The emerging importance of type I MADS box transcription factors for plant reproduction. Plant Cell.

[43] Li H.L., Wei L.R., Guo D., Wang Y., Zhu J.H., Chen X.T., Peng S.Q. (2016). HbMADS4, a MADS-box Transcription Factor from Hevea brasiliensis, Negatively Regulates HbSRPP. Front. Plant Sci..

[44] Liang M., Du Z., Yang Z., Luo T., Ji C., Cui H., Li R. (2024). Genome-wide characterization and expression analysis of MADS-box transcription factor gene family in Perilla frutescens. Front. Plant Sci..

[45] Han M., Lin S., Zhu B., Tong W., Xia E., Wang Y., Yang T., Zhang S., Wan X., Liu J. (2024). Dynamic DNA Methylation Regulates Season-Dependent Secondary Metabolism in the New Shoots of Tea Plants. J. Agric. Food Chem..

[46] Srikant T., Gonzalo A., Bomblies K. (2024). Chromatin Accessibility and Gene Expression Vary Between a New and Evolved Autopolyploid of Arabidopsis arenosa. Mol. Biol. Evol..

[47] Yang N., Li J.W., Deng Y.J., Teng R.M., Luo W., Li G.N., Hu Z.H., Liu H., Xiong A.S., Zhang J. (2024). Ectopic biosynthesis of catechin of tea plant can be completed by co-expression of the three CsANS, CsLAR, and CsANR genes. Hortic. Res..

[48] Rabier C.E., Ta T., Ané C. (2014). Detecting and locating whole genome duplications on a phylogeny: A probabilistic approach. Mol. Biol. Evol..

[49] Huang Y., Sahu S.K., Liu X. (2025). Deciphering recent transposition patterns in plants through comparison of 811 genome assemblies. Plant Biotechnol. J..

[50] Li X., Shen Z., Ma C., Yang L., Duan S., Lv Y., Yang L., Lei Y., Dong Y., Sheng J. (2023). Teabase: A comprehensive omics database of Camellia. Plant Commun..

[51] Suo A., Lan Z., Lu C., Zhao Z., Pu D., Wu X., Jiang B., Zhou N., Ding H., Zhou D. (2021). Characterizing microRNAs and their targets in different organs of *Camellia sinensis* var. *assamica*. Genomics.

[52] Xia E.H., Zhang H.B., Sheng J., Li K., Zhang Q.J., Kim C., Zhang Y., Liu Y., Zhu T., Li W. (2017). The Tea Tree Genome Provides Insights into Tea Flavor and Independent Evolution of Caffeine Biosynthesis. Mol. Plant.

[53] Chen J.D., He W.Z., Chen S., Chen Q.Y., Ma J.Q., Jin J.Q., Ma C.L., Moon D.G., Ercisli S., Yao M.Z. (2022). TeaGVD: A comprehensive database of genomic variations for uncovering the genetic architecture of metabolic traits in tea plants. Front. Plant Sci..

