# Supplementary material for: Epigenetic Regulation of Divergent Co-Expression Between Whole-Genome Duplication and Transposed Duplication Genes and Its Impact on Catechin Accumulation in Camellia sinensis
Source: Genes (Basel). 2026 Jul 30;17(8):898. doi: 10.3390/genes17080898 (PMC13512718; doi:10.3390/genes17080898)
Supplement: Supplementary file 1 [file genes-17-00898-s001.zip › S1.pdf]

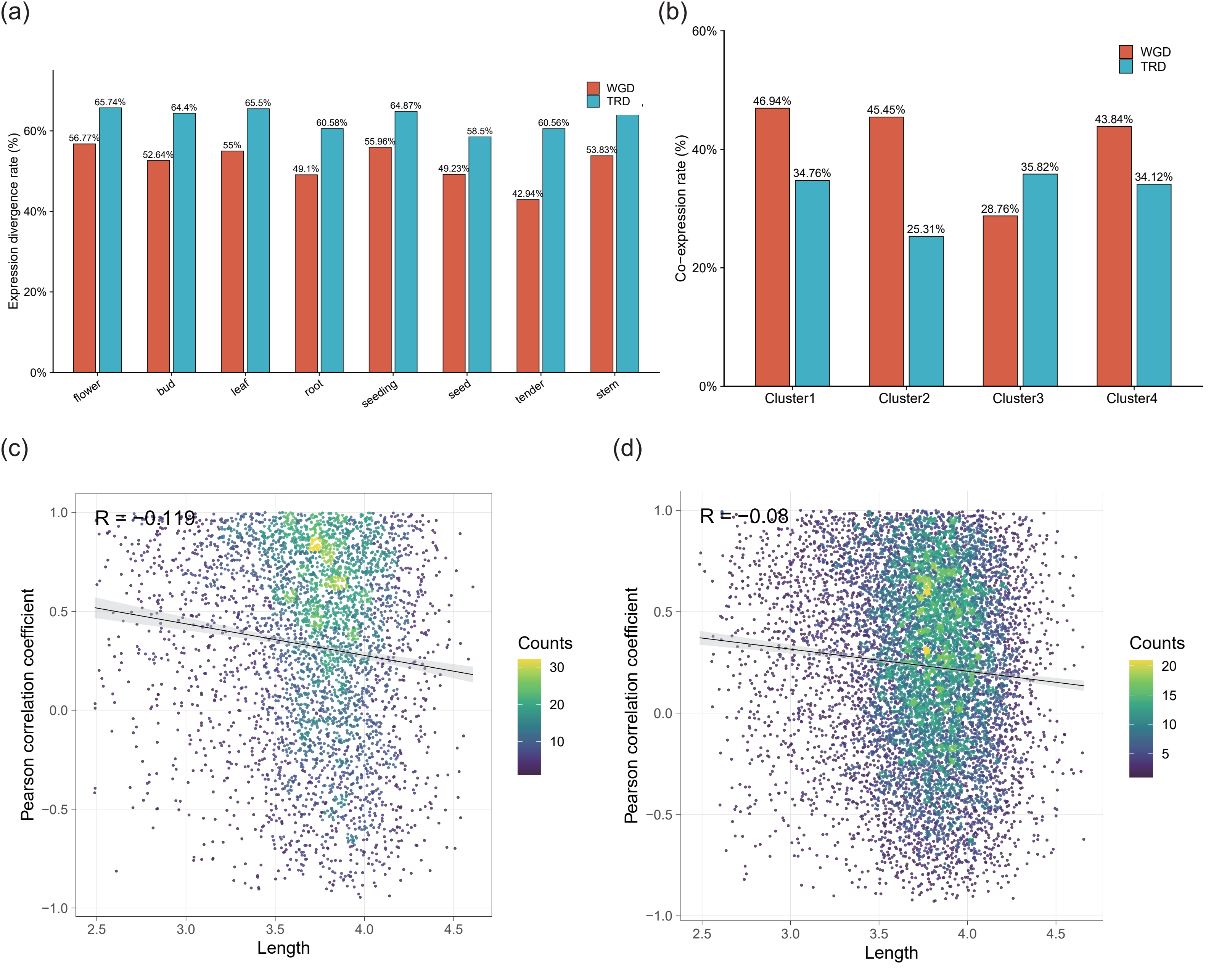

(a)
Expression divergence rate (%)
0%
20%
40%
60%
WGD
TRD
56.77%
65.74%
52.64%
64.4%
55%
65.5%
49.1%
60.58%
55.96%
64.87%
49.23%
58.5%
42.94%
60.56%
53.83%
flower
bud
leaf
root
seeding
seed
tender
stem
(b)
Co−expression rate (%)
0%
20%
40%
60%
WGD
TRD
46.94%
34.76%
45.45%
25.31%
28.76%
35.82%
43.84%
34.12%
Cluster1
Cluster2
Cluster3
Cluster4
(c)
R = −0.119
Pearson correlation coefficient
1.0
0.5
0.0
−0.5
−1.0
2.5
3.0
3.5
4.0
4.5
Length
Counts
30
20
10
(d)
R = −0.08
Pearson correlation coefficient
1.0
0.5
0.0
−0.5
−1.0
2.5
3.0
3.5
4.0
4.5
Length
Counts
20
15
10
5
