# Supplementary material for: Epigenetic Regulation of Divergent Co-Expression Between Whole-Genome Duplication and Transposed Duplication Genes and Its Impact on Catechin Accumulation in Camellia sinensis
Source: Genes (Basel). 2026 Jul 30;17(8):898. doi: 10.3390/genes17080898 (PMC13512718; doi:10.3390/genes17080898)
Supplement: Supplementary file 1 [file genes-17-00898-s001.zip › S2.pdf]

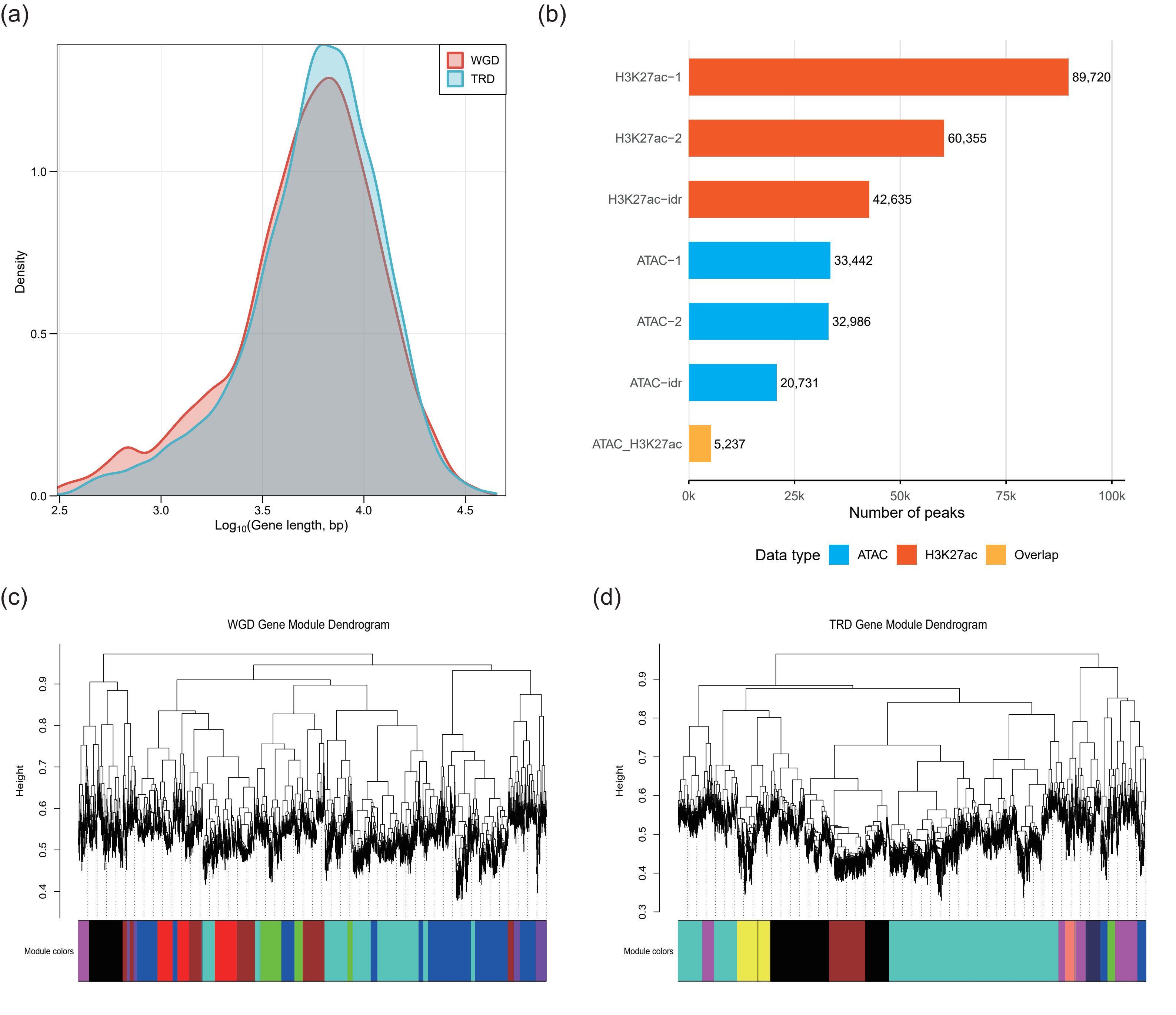

(a)
WGD
TRD
Density
1.0
0.5
0.0
2.5
3.0
3.5
4.0
4.5
Log10(Gene length, bp)
(b)
H3K27ac−1
89,720
H3K27ac−2
60,355
H3K27ac−idr
42,635
ATAC−1
33,442
ATAC−2
32,986
ATAC−idr
20,731
ATAC_H3K27ac
5,237
0k
25k
50k
75k
100k
Number of peaks
Data type
ATAC
H3K27ac
Overlap
(c)
WGD Gene Module Dendrogram
Height
0.9
0.8
0.7
0.6
0.5
0.4
Module colors
(d)
TRD Gene Module Dendrogram
Height
0.9
0.8
0.7
0.6
0.5
0.4
0.3
Module colors
