# Supplementary material for: Epigenetic Regulation of Divergent Co-Expression Between Whole-Genome Duplication and Transposed Duplication Genes and Its Impact on Catechin Accumulation in Camellia sinensis
Source: Genes (Basel). 2026 Jul 30;17(8):898. doi: 10.3390/genes17080898 (PMC13512718; doi:10.3390/genes17080898)
Supplement: Supplementary file 1 [file genes-17-00898-s001.zip › S3.pdf]

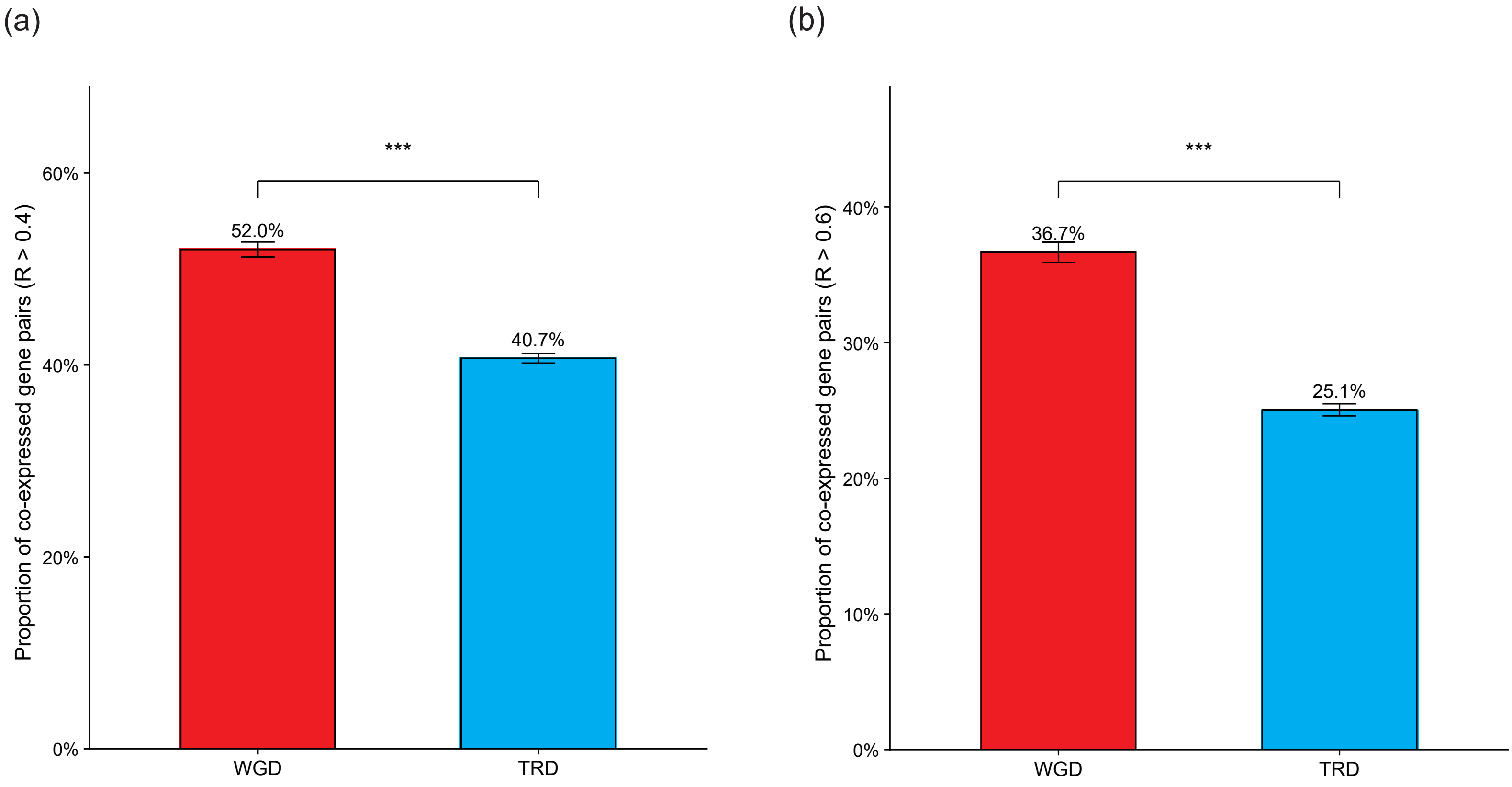
(a)
Proportion of co-expressed gene pairs (R > 0.4)
60%
40%
20%
0%
***
52.0%
40.7%
WGD
TRD
(b)
Proportion of co-expressed gene pairs (R > 0.6)
40%
30%
20%
10%
0%
***
36.7%
25.1%
WGD
TRD
